# Predictors of national health insurance membership among the poor with different education levels in Indonesia

**DOI:** 10.1186/s12889-023-15292-9

**Published:** 2023-02-21

**Authors:** Nuzulul Kusuma Putri, Agung Dwi Laksono, Nikmatur Rohmah

**Affiliations:** 1 Faculty of Public Health, Universitas Airlangga, Surabaya, Indonesia; 2 The Airlangga Centre for Health Policy (ACeHAP), Surabaya, Indonesia; 3 National Research and Innovation Agency, Republic of Indonesia, Jakarta, Indonesia; 4 Faculty of Health Science, Muhammadiyah University of Jember, Jember, Indonesia

**Keywords:** Education, National Health Insurance, Universal health coverage, Disparity, Social security

## Abstract

**Background:**

Indonesia has made significant progress in expanding universal health coverage (UHC) through its National Health Insurance (NHI) mechanism. However, in the context of NHI implementation in Indonesia, socioeconomic disparities caused its subpopulations to have different literacy of NHI concepts and procedures, increasing the risk of healthcare access inequities. Hence, the study aimed to analyse the predictors of NHI membership among the poor with different education levels in Indonesia.

**Methods:**

This study used the secondary dataset of the nationwide survey “Abilities and Willingness to Pay, Fee, and Participant Satisfaction in implementing National Health Insurance in Indonesia in 2019” by The Ministry of Health of the Republic of Indonesia. The study population was the poor population in Indonesia and included a weighted sample of 18,514 poor people. The study used NHI membership as a dependent variable. Meanwhile, the study analysed seven independent variables: wealth, residence, age, gender, education, employment, and marital status. In the final step of the analysis, the study used binary logistic regression.

**Results:**

The results show that the NHI membership among the poor population tends to be higher among those who have higher education, live in urban areas, are older than 17 years old, are married and are wealthier. The poor population with higher education levels is more likely to become NHI members than those with lower education. Their residence, age, gender, employment, marital status, and wealth also predicted their NHI membership. Poor people with primary education are 1.454 times more likely to be NHI members than those without education (AOR 1.454; 95% CI 1.331–1.588). Meanwhile, those with secondary education are 1.478 times more likely to be NHI members than those with no education (AOR 1.478; 95% CI 1.309–1.668). Moreover, higher education is 1.724 times more likely to result in being an NHI member than no education (AOR 1.724; 95% CI 1.356–2.192).

**Conclusion:**

Education level, residence, age, gender, employment, marital status, and wealth predict NHI membership among the poor population. Since significant differences exist in all of those predictors among the poor population with different education levels, our findings highlighted the importance of government investment in NHI, which must be supported with investment in the poor population’s education.

## Introduction

Poverty is a state of being unable to meet basic needs such as food and non-food [1–3]. A population is categorised as poor if it has an average monthly per capita expenditure below the poverty line [1]. Economic conditions negatively influence poverty; the better the economy, the lower the poverty will be [4]. Poverty increases the risk of death and disability due to non-communicable diseases (NCDs) [5]. On the other hand, NCDs also increase the risk of falling into poverty [6]. Illness also causes heavy expenses along with the experience of physical and mental suffering. Usually, in countries without social health insurance, an unbearable amount of medical costs is incurred to improve or maintain the patient’s health condition. As a result, heavy financial burdens tend to push households from a comfortable or secure life, or even from bad to worse, to the possibility of becoming poor [7]. Poor people are vulnerable people. Without health insurance, they fall into deeper poverty once they become sick.

In Indonesia, the National Health Insurance (NHI) is a form of government’s commitment to achieving universal health coverage (UHC). NHI was first established based on the 2004 National Social Security System (NSSS) Law and was first implemented in 2014. Before NHI implementation, Indonesian citizens financed their healthcare costs through out-of-pocket payments, and only citizens listed as poor in the government database were covered by their home district health insurance. The local government financed this district health insurance with different health benefits based on their fiscal capacity. The poor who were not listed in the database or lived in a district where the local government did not provide health insurance had to pay their own healthcare cost. For that reason, NHI is expected to help low-income individuals to cover their out-of-pocket healthcare cost, which is often difficult to predict and usually entails very high prices.

The 2004 NSSS Law states that everyone living in Indonesia must have coverage under the NHI regardless of being poor [8]. All citizens must contribute to NHI by paying a monthly fixed premium to cover the cost of health services that may arise when they are sick [9]. Under the NHI, all existing district health insurance and other social assistance to pay for the poor’s healthcare were merged and used to pay the NHI premium. Hence, the poor covered by the NHI are called Contribution Assistance Recipients (CAR). NHI is expected to cover all Indonesian people equally, supporting health equity for the poor and near-poor in Indonesia [10].

Even though NHI targeted membership coverage of 95% by the end of 2019, it only achieved 85.3% [8]. The poor population is more likely to enroll in the NHI than other members who must pay the NHI premium [11, 12]. Studies in Ghana and Benin that also used national health insurance and exemptions for the poor in paying NHI premiums revealed that the core poor are excluded from the NHI because this population does not understand that they have the right to be funded and does not know the procedures to acquire their privilege as the poor in NHI [13–15]. In Indonesia, individuals unable to understand and fulfil the registration procedure tend not to have a membership, while individuals with better access to information have more benefits in understanding the NHI [16–18]. Studies found that the poor living in an urban area, where the NHI information is easily accessed, are reportedly more likely to be enrolled [11, 19] as were the poor who accessed the internet [20–22].

In addition, education is significantly essential in determining individual membership in the NHI. Previous national data studies prove that education relates to community participation status in the NHI [11, 23]. Studies also support that in the regions with different characteristics, such as the far inland of Singkil, the urban setting in Manado, and the rural setting in Bojonegoro, education is crucial in predicting NHI membership [17, 24]. The low-education population tends not to understand the eligibility and requirement to be registered as CAR [16]. It explained why many citizens included in the poverty indicators, according to Indonesia Statistics, had not yet registered as CAR [25, 26]. The education level is also related to community participation in independent NHI, intended for citizens working in informal sectors [27, 28]. Low education has become a barrier for informal workers to understand the self-registered mechanism, the NHI benefits package, and how to use NHI [17, 27]. Based on the background research, the study aimed to analyse the predictors of NHI membership among the poor with different education levels.

## Materials and methods

### Data source

The study used secondary data from a nationwide survey, “Abilities and Willingness to Pay, Fee, and Participant Satisfaction in implementing National Health Insurance in Indonesia in 2019,“ performed by The Ministry of Health of the Republic of Indonesia. The study population is poor people in Indonesia. The Ministry of Health does not publicly publish the data and final report on its website, but the public can access it based on request.

The survey used the wealth index formula to determine wealth status. The wealth index is a weighted measure of the total spending of a household. Meanwhile, the survey calculated the wealth index using primary data on household spending on health insurance, food, accommodation, and other items. Furthermore, the pool divided the income index into five quintiles in the poll: quintile 1 (the poorest), quintile 2 (poorer), quintile 3 (middle), quintile 4 (richer), and quintile 5 (the richest) [29, 30]. In this study, the poor are quintiles 1 and 2. The study described 18,514 weighted poor people as research respondents through stratification and multistage random sampling. This nationwide study involved 715 districts, 1430 villages, and 14,300 households in all Indonesian provinces (34 provinces).

The data were collected through an offline survey from March to December 2019. The study used household and individual instruments to assess participant characteristics, health insurance ownership, and NHI membership.

### Outcome variable

The outcome variable in the study was participation in the National Health Insurance (NHI) membership. NHI membership refers to the respondent’s involvement in the NHI, whether as an individual member, a required member (civil servant, police officer, or army), borne by the company, or a Contribution Assistance Recipient (CAR). NHI membership comes in two varieties: non-member and member.

### Exposure variable

The study utilised seven factors as exposure variables: education level, type of residence, age group, gender, employment status, marital status, and wealth status. We divided the education level into four categories: none, primary, secondary, and higher. Furthermore, there are two options for housing: urban and rural. The age comprises 17 or less, 18–64, and 65 or more. The study divided gender into two categories: male and female. Employment status includes unemployed and employed. Furthermore, marital status contains three types: never married, married, and divorced/widowed. The study splits wealth status into the poorest and poorer.

### Data analysis

As for comparing other exposure variables at the education level, the authors performed a bivariate comparison by the Chi-square test in the initial step. Furthermore, a collinearity test was used in the study to guarantee that there was no strong correlation between independent variables in the final regression model. The authors applied a binary logistic regression in the study’s last point. This test was performed as part of the survey to investigate the multivariate connection between all independent variables and NHI membership as the dependent variable. The authors employed the IBM SPSS 26 application for statistical analysis.

On the other hand, using the same data, the study employed ArcGIS 10.3 (ESRI Inc., Redlands, CA, USA) to map the distribution of NHI membership among the poor in Indonesia. The Indonesian Bureau of Statistics provided a shapefile of administrative border polygons for the study.

## Results

**Fig. 1:**
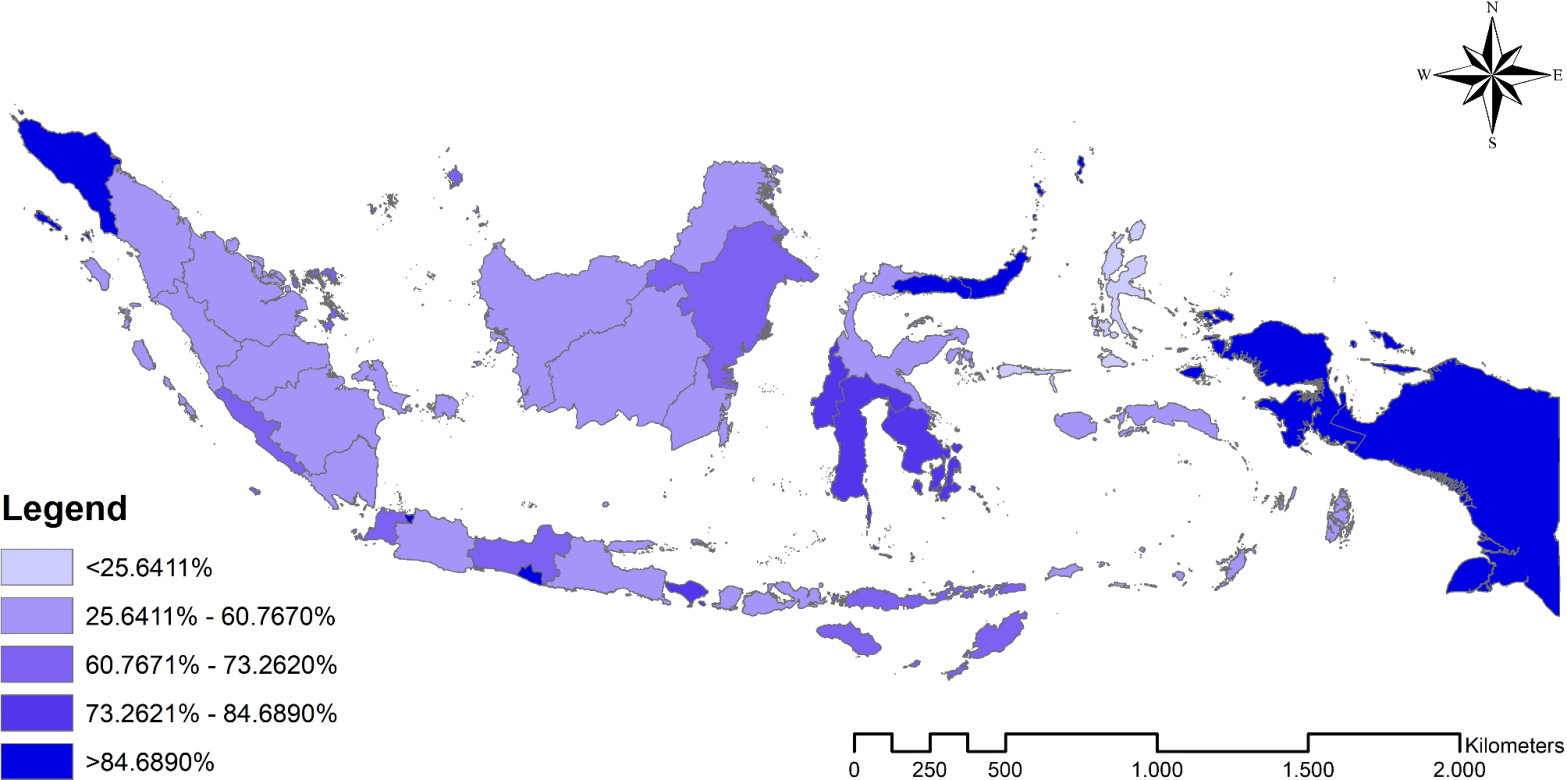
The Distribution Map of NHI membership among the poor in Indonesia

The study found that the average NHI membership among the poor in Indonesia was 65.1%. Meanwhile, the distribution of education levels for the poor in Indonesia is as follows: no education (16.9%), primary education (64.1%), secondary education (16.9%), and higher education (2.2%). Figure 1 shows the trend of the highest proportion of NHI membership among the poor at the western and eastern ends. Moreover, Table 1 displays descriptive statistics of NHI membership among the poor in Indonesia.

Single-factor analysis was used to investigate the influence of education level on the other observed predictors of NHI membership. Table 1 shows the distribution of the poor by residence, age, gender, employment, marital status, and wealth differed in education levels (p < 0.0000). Therefore, significant differences exist in NHI membership predictors among poor people with different education levels.

Table 1 acknowledges a difference between NHI members in their education levels. The higher the level of education, the higher the proportion of the poor who are NHI members. According to the type of residence, the higher the level of education, the lower the proportion of the poor living in rural areas. Based on age group, the higher the level of education, the lower the proportion of the poor in the ≥ 65. Based on gender, females dominated all education levels, except in secondary education, where males headed the group. Regarding employment status, the employed lead in all education categories, except no education, and the unemployed lead in the class. Meanwhile, according to marital status, married led all education groups, except no education among those who never married. Moreover, based on wealth status, the poorer dominated in all education categories except primary education, which the poorest led.

**Table 1:** Descriptive statistics of NHI membership among the poor in Indonesia (n = 18,514)

Characteristics	Education Level				p-value
	No education (n = 3,157)	Primary (n = 11,782)	Secondary (n = 3,187)	Higher (n = 388)	
**NHI membership**					< 0.001
Non-member	45.2%	33.5%	30.8%	27.2%	
Member	54.8%	66.5%	69.2%	72.8%	
**Type of residence**					< 0.001
Urban	13.9%	15.6%	26.2%	26.2%	
Rural	86.1%	84.4%	73.8%	73.8%	
**Age group**					< 0.001
≤ 17	64.6%	20.6%	2.1%	0.0%	
18–64	22.7%	67.6%	94.7%	93.0%	
≥ 65	12.8%	11.8%	3.2%	7.0%	
**Gender**					< 0.001
Male	46.8%	48.2%	55.8%	42.4%	
Female	53.2%	51.8%	44.2%	57.6%	
**Employment status**					< 0.001
Unemployed	76.8%	46.8%	37.8%	24.2%	
Employed	23.2%	53.2%	62.2%	75.8%	
**Marital status**					< 0.001
Never married	67.0%	29.9%	41.9%	36.3%	
Married	22.1%	59.1%	51.7%	54.7%	
Divorced/Widowed	10.9%	11.0%	6.4%	9.0%	
**Wealth status**					< 0.001
Poorest	49.7%	52.0%	44.6%	38.2%	
Poorer	50.3%	48.0%	55.4%	61.8%	

The study performed a collinearity test in the following analysis. The research findings show no strong relationship between the independent variables. The tolerance value for all variables is more significant than 0.10, and the variance inflation factor (VIF) value for all variables is less than 10.00. The study can state that there are no indicators of multicollinearity in the regression model.

Table 2 shows the result of the binary logistic regression of NHI membership in Indonesia. Based on education level, those having primary education are 1.454 times more likely to be an NHI member than those with no education (AOR 1.454; 95% CI 1.331–1.588). Meanwhile, those having secondary education are 1.478 times more likely to be NHI member than those with no education (AOR 1.478; 95% CI 1.309–1.668). Moreover, those having higher education are 1.724 times more likely to be an NHI member than those having no education (AOR 1.724; 95% CI 1.356–2.192). The results indicate that the better the education level, the higher possibility of being an NHI member among the poor in Indonesia.

In addition to education level, the study also found four exposure variables related to NHI membership among the poor in Indonesia. Regarding the type of residence, the poor in rural areas are 0.733 times less likely to be NHI members than those in urban areas (AOR 0.733; 95% CI 0.673–0.798). According to age groups, all groups are more likely than those 17 or less to be members of NHI. Regarding marital status, the married poor are 0.877 times less likely to become an NHI member than those who never married (AOR 0.877; 95% CI 0.793–0.969). Furthermore, based on wealth status, Table 2 informs that the poorer are 1.199 times more likely to be an NHI member than the poorest (AOR 1.199; 95% CI 1.126–1.277).

**Table 2:** The result of binary logistic regression of NHI membership among the poor in Indonesia (n = 18,514)

Predictor	Member of NHI	
	AOR (95% CI)	p-value
Education: No Education (Ref.)		
Education: Primary	1.454 (1.331–1.588)	*<0.001
Education: Secondary	1.478 (1.309–1.668)	*<0.001
Education: Higher	1.724 (1.356–2.192)	*<0.001
Residence: Urban (Ref.)		
Residence: Rural	0.733 (0.673–0.798)	*<0.001
Age group: ≤ 17 (Ref.)		
Age group: 18–64	1.538 (1.360–1.738)	*<0.001
Age group: ≥ 65	1.827 (1.557–2.144)	*<0.001
Gender: Male (Ref.)		
Gender: Female	1.011 (0.945–1.082)	0.756
Employment: Unemployed (Ref.)		
Employment: Employed	0.941 (0.868–1.019)	0.135
Marital: Never married (Ref.)		
Marital: Married	0.877 (0.793–0.969)	**0.010
Marital: Divorced/Widowed	1.078 (0.932–1.247)	0.314
Wealth: Poorest (Ref.)		
Wealth: Poorer	1.199 (1.126–1.277)	*<0.001

Note: AOR: Adjusted Odds Ratio; CI: confidence interval; *p < 0.001; **p < 0.050

## Discussion

Embracing government decentralisation, the central government pays for the poor’s contribution to the NHI. The Ministry of Social Welfare updates its list of poor people nationwide annually, and only people on that list will be eligible to become CAR. When any population cannot be considered as the CAR the central government funds, they still have the opportunity to be funded under the local government’s budget. The local government will enlist them as NHI participants based on the individual’s self-report that they are poor and need financial aid to access healthcare services. This self-reported procedure involves complex steps of verification and document requirements by several local government offices.

The formal education level among the poor in our study predicts the poor’s enrolment in NHI. It shows that even in a compulsory social health insurance scheme like the NHI, where the government determines the poor’s enlistment for eligibility, health insurance literacy is still significantly critical. Our study found that the better the formal education level, the higher possibility of being an NHI member among the poor in Indonesia. The poor people’s identification to be a CAR needs a bureaucratic procedure that not only requires the poor to fulfil legal documents and meet the authorised offices but also needs an understanding of how the system works. People with low education levels will find this more complicated than those with higher education levels. The poor with low education level are not only vulnerable to not being covered by the NHI, but those who are already enrolled have low hospital utilisation since they do not understand how to use the NHI to access the hospital [19, 31]. This finding is consistent with other studies in other low-middle-income countries. In Chad and Ghana, those who never had a formal education are less likely to be covered by health insurance even though their countries provide a social health insurance scheme (15). Formal education level is often associated with literacy [20, 32]. Health insurance literacy, the ability to understand what health insurance is, is a prominent factor in health insurance participation [18]. A proper understanding of health insurance will lead to a higher acceptance rate for participating in health insurance [20].

Before the NHI, Indonesia funded its poor population through different social health insurance scenarios that varied between districts. Those social health insurance scenarios merged into the NHI, disrupting how poor people can be covered with health insurance. Before the NHI was first implemented in 2014, the poor covered by the district health insurance were not automatically changed into NHI members. The districts were still calculating to enrol all their poor citizens in the NHI based on their fiscal capacity. Hence, this dynamic change triggered uncertainty among the poor. The lower education population could have difficulty understanding the abrupt and complex change in the NHI mechanism [11, 16, 32]. Higher education and adequate information are two things that are always related to higher health insurance literacy [16, 18, 32]. Thus, it is not surprising that, in several studies, people’s exposure to information sources, such as mass media, is also reported to increase the probability of people with no education background enrolling in a health insurance coverage [15, 33]. A lack of accurate information about the NHI and the ability to understand that information may cause more of the poor population to fail to realise what the NHI’s benefit is and how they can be enlisted as NHI participants [16].

Our study informs that the poor in rural areas are less likely to be NHI members than those in urban areas. The population in a rural area in Indonesia is commonly referred to as a subpopulation with limited information access because of the limited internet infrastructure but also its expensive connection [34]. In respect that Indonesia is an archipelago with more than a thousand inhabited islands, its dispersed geographic condition not only becomes a significant problem in the doctors’ distribution [35] but also leads to unequal distribution of health information [36]. On the other hand, the internet plays a vital role in persuading people to participate in the NHI [22]. It shares updated information on the NHI and its interaction among the population, influencing others to decide their participation in the NHI [22, 37]. Compared to the poor people in the urban area, even though they acknowledged that the procedures for accessing social health insurance provided by the government are complicated, they have more possibility to access health information so that they know how to be able to enlist in the CAR [38].

Age is likely a barrier to becoming an NHI member in Indonesia. Even though other studies reported different age groups that correlate with low participation rates in the NHI [11, 16, 23, 27], most studies consistently report that younger people have less probability of being enlisted as NHI participants. Our study shows that all groups are likelier than those seventeen years old or younger to be members of NHI. Other studies report that the elderly (≥ 65 years old) have the highest likelihood of NHI membership among all age groups [11, 23]. Willingness to pay is mainly used to explain how the age group could become a predictor of health insurance coverage [39–41]. However, since our study focuses on the poor, for whom the government pays their contribution to the NHI, willingness to pay could not be used to explain why the younger population has a lower likelihood of being an NHI member. We argue that it is related to health insurance literacy among the poor. In the NHI, the member enrolment must be submitted as a household unit, meaning that the head of household must report all of their family members to become eligible for CAR. The education of the household head is significantly associated with their family members’ enrolment in health insurance. Household heads with higher secondary education or above have a higher probability of insurance coverage than household heads with no formal education [33].

Our study also found that the married poor are less likely to become NHI members than those who never married. A single, never-married population does not have family members who need to be enrolled as NHI participants [32, 39]. It indicates that NHI membership could not merely be associated with the population’s willingness to pay. At this point, the education level could become a prominent predictor. Considering that the never-married group is mainly at a productive young age, many studies claim that the low health insurance membership among the never-married population is highly related to their perception of health-related risk [11, 23, 32, 39]. Younger people commonly think they are healthy enough, so they do not have any urgency to be covered by health insurance. Health-related risk perception is essential in motivating health behaviour change [42]. Insufficient risk communication about the importance of health insurance reportedly leads to low demand for health insurance since people do not have any encouragement to manage health risks by enrolling in a health insurance scheme (43).

Lastly, we also find that the poorer in our study are more likely to be NHI members than the poorest. Wealthier populations are likely to use private health facilities due to the healthcare quality provided because they have greater ability to pay for the insurance premium [15, 33]. However, since our study focuses only on the poor population in a social health insurance scheme, the same reason cannot explain why the poorest have a lower probability of being enrolled in the NHI than the poorer. As the lowest quintile of the wealthy population, the poorest suffer from various wealth-related inequalities. Observed disparities in education, media exposure, and the geographical location of their residence as the wealth-related inequality in our study explain why the poorest are less likely to become NHI members than the poorer. Further, several studies in the Indonesian context reported that low education levels are attributed to the poor population, which then makes the poor not have sufficient health insurance literacy [11, 23]. Our findings strengthen other studies which reported that low-and middle-income countries, which generally use NHI as their primary strategy to achieve UHC, fail to protect their underserved populations and predominantly support better-off population groups [44].

### Study limitation

This study has the advantage of using big data as an analysis material, allowing the conclusions to be extrapolated to the national level. On the other hand, the analysis in this study is based on secondary data. The variables considered are limited to those provided by the Republic of Indonesia’s Ministry of Health. Several other variables previously known to influence health insurance ownership could not be investigated. These variables include cognitive capacity, prior commercial insurance ownership, having children, and family size [45–47].

## Conclusion

This study concluded that NHI membership among the poor population could be predicted based on their education level, residence, age, gender, employment, marital status, and wealth. All those predictors are different between the education levels of poor people. The better the education level, the higher possibility of being an NHI member among the poor in Indonesia. The education disparities in this study explain unequal opportunities and access, which hinder the poor population covered by health insurance using government aid. The education level of the poor predicts their health insurance literacy, which determines their ability to claim their right to become a CAR.

Based on the conclusions, the authors recommend that the government ensure social inclusion in their mechanism, enlisting the poor eligible to be CAR. It must meet the needs of the poor who do not have enough ability to understand complex procedures in accessing the NHI. In addition, the NHI promotion message must be able to explain the procedures and health-related risks to initiate the poor’s willingness to claim their right to be funded by the government. Hence, it is expected that government aid can and does provide direct benefits to the poor and promotes their health and well-being. Our findings highlighted the importance of government investment in NHI, which must be supported with investment in the poor population’s education.

## Data Availability

The datasets used and/or analyzed during the current study are available from the corresponding author upon reasonable request.
